# The Analysis of Emotion Authenticity Based on Facial Micromovements

**DOI:** 10.3390/s21134616

**Published:** 2021-07-05

**Authors:** Sung Park, Seong Won Lee, Mincheol Whang

**Affiliations:** 1 School of Design, Savannah College of Art and Design, Savannah, GA 31401, USA; 2 Department of Human Centered Artificial Intelligence, Sangmyung University, Jongno-gu, Seoul 03016, Korea; wonii0916@gmail.com (S.W.L.); whang@smu.ac.kr (M.W.)

**Keywords:** facial micromovement, emotion recognition, emotion authenticity

## Abstract

People tend to display fake expressions to conceal their true feelings. False expressions are observable by facial micromovements that occur for less than a second. Systems designed to recognize facial expressions (e.g., social robots, recognition systems for the blind, monitoring systems for drivers) may better understand the user’s intent by identifying the authenticity of the expression. The present study investigated the characteristics of real and fake facial expressions of representative emotions (happiness, contentment, anger, and sadness) in a two-dimensional emotion model. Participants viewed a series of visual stimuli designed to induce real or fake emotions and were signaled to produce a facial expression at a set time. From the participant’s expression data, feature variables (i.e., the degree and variance of movement, and vibration level) involving the facial micromovements at the onset of the expression were analyzed. The results indicated significant differences in the feature variables between the real and fake expression conditions. The differences varied according to facial regions as a function of emotions. This study provides appraisal criteria for identifying the authenticity of facial expressions that are applicable to future research and the design of emotion recognition systems.

## 1. Introduction

Humans utilize both verbal and nonverbal communication channels. The latter category includes facial expressions, gestures, posture, gait, gaze, distance, and tone and manner of voice [1]. Facial expressions, which account for up to 30% of nonverbal expressions, are the most rapidly processed type of expression by visual recognition [2]. Facial expressions project the communicator’s intentions and emotions [3]. However, people may conceal their true feelings and produce fake expressions [4]. Such false expressions are exhibited for a very short time with only subtle changes [5], and it is extremely difficult to detect their authenticity with eyesight [6]. Identifying fake expressions is paramount to counter deception and recognize users’ true intent in advanced intelligent systems (e.g., social robots and assistive systems).

Early research involving facial expressions focused on establishing a quantitative classification framework to recognize emotions. Ekman built a facial action coding system (FACS), a computation system that encodes facial features’ movements to taxonomize emotions from facial expressions. Analysis of facial expressions also spurred interest in the authenticity of expressions.

Researchers have found asymmetric intensity in facial expressions. Dopson revealed that the intensity of expressions in the left face was stronger than that in the right face in the case of voluntary expressions [7]. Conversely, the intensity was weaker than that in the right face in the case of involuntary expressions. These results suggest that the comparison of both sides may identify the authenticity of expressions. The sensitivity of left-face expressions is because facial movements are connected to the right hemisphere of the brain. Patients with right-brain injuries are reported to experience significant degradation in recognizing emotions from facial expressions compared with patients with left-brain injuries [8].

Studies have also found differential activation of facial muscles between real and fake expressions. Duchenne experimented on facial muscular contractions with electrical probes to understand how the human face produces expressions [9]. He observed that participants produced a genuine smile with a unique contraction of the Orbicularis oculi muscle [10]. This “smiling with the eyes” is called the Duchenne smile, in his honor.

Ekman analyzed human false expressions and identified minute vibrations or spontaneous changes in the facial muscles responsible for emotional expression [11]. Such micromovements are observed in false (e.g., deception) or pretended (e.g., to be polite) expressions [5]. Facial micromovement is also called microexpression. Micromovement occurs with less than a second of movement and with vibration lasting between 0.04 and 0.5 s [12,13,14]. Simultaneously, in a typical interaction, an emotional expression begins and ends with a macroexpression that occurs in less than 4 s [15]. The degree of movement or the vibration of the facial muscles between real and fake expressions can be significantly different [11].

Recent advances in AI technology have led to research on identifying the authenticity of facial expressions using repetitive training with paired data of facial expressions and visual content (an image and a video clip) [16,17]. Microexpression recognition (MER) researchers have put massive effort into open innovation (e.g., facial microexpressions grand challenge [18,19]) to improve the state-of-the-art algorithm. Academic challenges include all aspects of MER sequences such as data collection, preprocessing (face detection and landmark detection), feature extraction, microexpression recognition, and emotion classification within the computer vision domain (for a comprehensive review, see [20] and [21]). Similar to other AI domains, convolutional neural networks (CNNs) have been used the most for MER [22]. A generative adversarial network (GAN), with a generator and an adversarial discriminator model, has been used for feature extraction [23] and facial image synthesis [24]. Most recently, extended local binary patterns on three orthogonal plans (ELBPTOP) were introduced to counter information loss and computational burden of the previous dominant descriptors, LBPTOP [25].

While researchers continue to pursue better algorithms to improve MER accuracy and reliability, in the most recent survey of facial microexpression analysis [20], Xie observed that MER literature on facial asymmetrical phenomena is scarce and limited. While researchers have found an asymmetric intensity in facial expressions, less is known regarding where in the facial region such microexpressions are the most salient and how they interact with different emotions. Specifically, feature variables (i.e., the degree and variance of movement, and vibration level) of emotions that are primarily expressed with the relaxation of facial muscles (e.g., contentment, sadness) may have weaker intensity in the real condition. Systematic research identifying reliable indicators of authenticity per facial region as a function of emotion is imperative.

Physiological data, including electrocardiogram (ECG), are powerful signals for emotion identification [26]. ECG correlates with the contraction of the heart muscles and varies as a function of emotion [27]. In order to achieve a deeper understanding of MER, facial vision data should be fused with cardio signals [20]. To the best of our knowledge, no research has combined the two.

In summary, the study hypothesized that (1) there is a significant difference in the micromovements at the onset of expression between real and fake conditions, and (2) such differences vary by representative emotions (happiness, sadness, contentment, anger). The findings were cross-validated with neurological measurements (ECG).

## 2. Methods

### 2.1. Experiment Design

The present study used a 2 × 4 within-subject design. The authenticity factor had two levels (real and fake), and the emotion factor had four levels (happiness, sadness, anger, and contentment). The visual stimulus consisted of a still photo and a video clip. The still photo depicted a facial expression of the target emotion. The video clip, which was shown after the still photo, was a recording that was designed to induce either the target emotion shown in the still photo or a neutral emotion.

The participants were then asked to produce a facial expression that the participant felt while watching the still photo. The real condition was manipulated by showing the two materials, the still photo and the video clip, congruently. The false condition was manipulated by having the video clip induce a neutral emotion. In this case, participants were forced to produce a facial expression based on the photo that they viewed earlier. If a different emotion was induced other than neutral, the participant’s emotion may have been compounded, which made the measurements difficult to explain. After every 30 s during the video, participants were signaled with a visual cue to produce a facial expression.

The dependent measurements involved micromovements in the face. That is, the average movement, standard deviation, and variance of the facial muscle movements were measured. Facial vibration was analyzed with the dominant frequency elicited by the fast Fourier transform (FFT).

### 2.2. Participants

Fifty university students were recruited as participants. The participants’ average age was 22.5 years (SD = 2.13) with an even ratio in gender. We selected participants with corrective vision of 0.7 or above to ensure the participants’ reliable recognition of visual stimuli. The participants were not allowed to wear glasses. All participants were briefed on the purpose and procedure of the experiment and signed a consent form. Participants were compensated with participation fees.

### 2.3. Procedure and Materials

**Figure 1:**
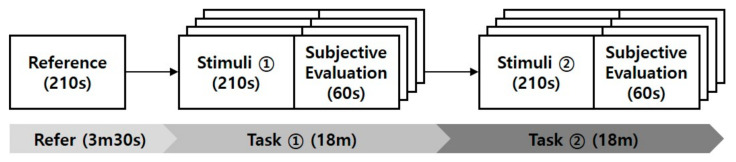
Experimental procedure.

Figure 1 illustrates the experimental procedure. Each participant’s neutral facial expression was captured for 210 s before the main task. This was considered as the individual’s reference expression. Participants were then exposed to eight combinations of visual stimuli—four sets (happiness, sadness, anger, and contentment) to elicit real emotions and four sets to elicit fake emotions. The order was randomized to counter order and learning effects. A set of stimuli consisted of a still photo and a video clip. A set used to induce real emotion had congruent emotions between the two materials. Conversely, a set to induce false emotions had inconsistent emotions between the two materials. In this case, the video clip induced a neutral emotion.

After viewing the visual stimuli, the participants were given a resting period of 60 s. During this period, participants reported their current emotional state with a subjective evaluation. Participants reported their (1) emotional state (happiness, sadness, anger, disgust, fear, surprise, and contentment), (2) degree of arousal, and (3) degree of pleasantness. The latter two were rated on a five-point Likert scale. (1) We provided a comprehensive set of seven emotions to select from to exclude any data from participants who felt nothing or had a different emotion from the target emotion. The exclusion was determined for each condition, even for neutral video clips, to eliminate any compounding factors from the data.

The participant’s facial data were acquired using a webcam. A Logitech c920 webcam (Logitech, Lausanne, Switzerland) was used to obtain image data with a resolution of 1280 × 980 at 30 frames per second. To analyze the activation level of the autonomic nervous system (ANS) when participants were exposed to visual stimuli, participants’ heart rate variability (HRV) and electrocardiogram (ECG) data were acquired. The latter was obtained through a Biopac (Biopac, Goleta, CA, USA) system with a frequency of 500 Hz.

**Figure 2:**
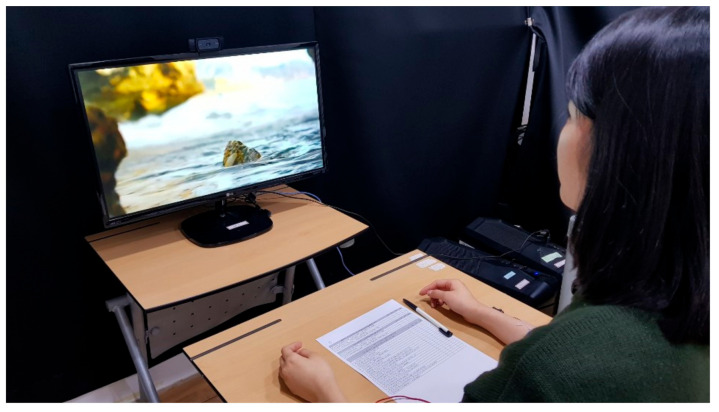
Experimental setting.

Figure 2 shows the experimental setup. Participants were asked to sit and view the experiment monitor at a distance of 60 cm. A webcam, which acquired facial data from the participant, was placed on top atop the monitor.

### 2.4. Statistical Analysis

The present study compared the differences in the micromovement of facial expressions between real and fake emotions. From the participant’s expression data, feature variables (i.e., the degree and variance of movement, as well as vibration level) obtained at 4 s (macromovement), 1 s, and 0.5 s (micromovements) after the onset (*t*) of facial expression were analyzed. For each representative emotion (happiness, contentment, anger, and sadness), a *t*-test was used to compare the differences between the feature variables in the two conditions (real and fake) for all 11 AUs responsible for emotional expression. The following section explains how the feature variables were extracted and how the ECG data were obtained.

## 3. Analysis

**Figure 3:**
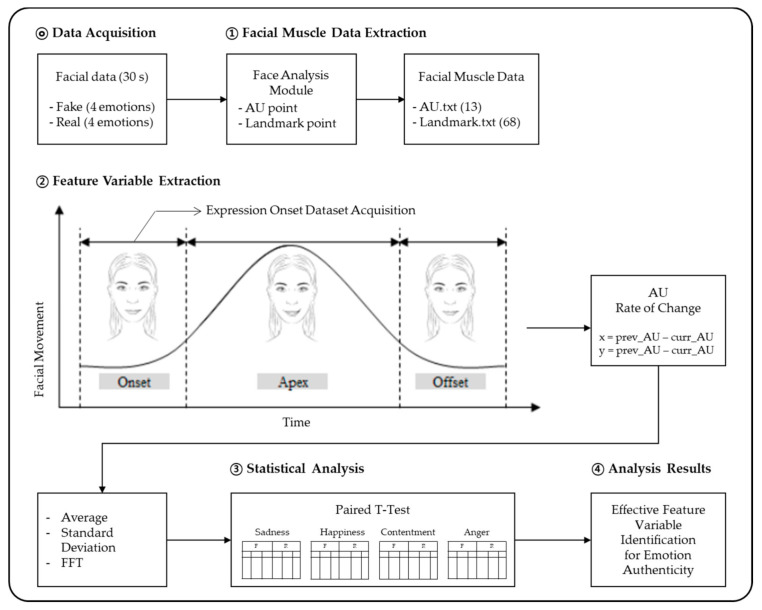
Data analysis process.

Figure 3 outlines the analysis process. To analyze the data, we established an operational definition of facial expression muscles and extracted facial movement data for such muscles. In total, 40 datasets were analyzed; participants who had excessive facial movements or participants who did not display emotion were excluded. That is, the experimenter screened each recorded video clip and excluded participants who had turned their faces, clearly looking at an object outside of the screen, or when the system had failed to track their faces. To minimize the exclusion, we had instructed the participants to reduce the facial movement and look straight ahead.

The expression onset segment was defined (② in Figure 3) based on the threshold of facial movement. Feature variables were then extracted by comparing the rate of change in action units (AUs) between data frames. The effective feature variables were selected by comparing the feature variables of real and fake expressions for each emotion.

### 3.1. Operational Definition of Facial Muscles

**Figure 4:**
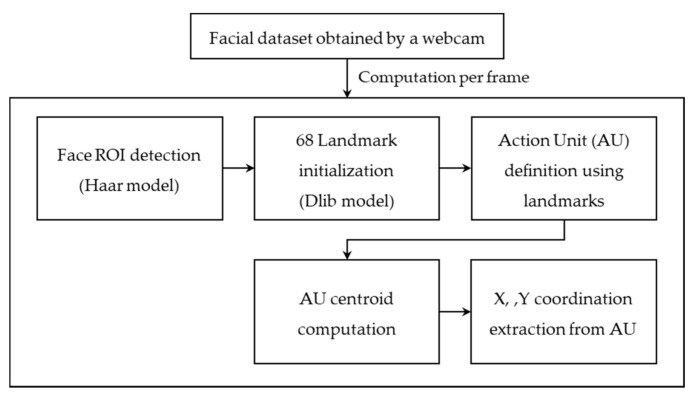
Facial muscle extraction process.

The present study recognized the participants’ emotions by identifying the activation of anatomical regions that represent a particular emotion. The AUs were extracted using facial landmarks through a Python program. Figure 4 depicts the extraction process.

**Figure 5:**
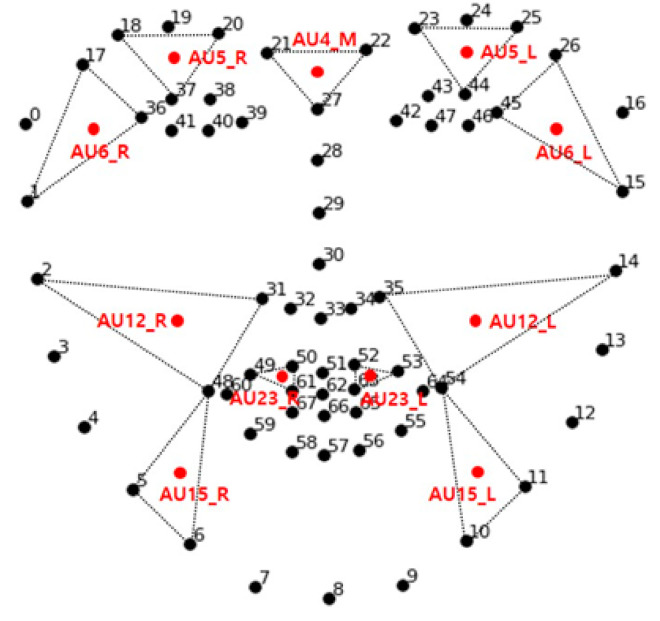
The relative locations of action units.

Each frame obtained from the webcam was analyzed. First, the location of the face in the image was identified using a face detection model, the Haar cascade classifier [28]. Face detection models extracted the target object’s features from the dataset and compared the features from the pretrained data to identify the object. Specifically, the present system used the Haar-like feature to detect the region of a face (region of interest (ROI)) by identifying the location of the nose and eyes. The system then identified 68 facial landmarks by tracking the eye, eyebrows, nose, lips, and chin line using the Dlib library [29], which was trained with a massive quantity of data. The differential facial muscles per facial expression were predefined and utilized to extract 11 muscle areas (i.e., coordination). Eleven facial muscle units (AUs) involving the brow, eyes, cheeks, chin, and lips responsible for facial expressions were predefined and extracted from the participant’s dataset (see Table 1). Figure 5 visualizes the relative locations of action units.

These 11 AUs are the centroid values of the three corresponding facial landmarks, computed as follows:

$$\begin{array}{l} A(x_{1},y_{1}),B(x_{2},y_{2}),C(x_{3},y_{3})\\ P(\frac{x_{1}+x_{2}+x_{3}}{3},\frac{y_{1}+y_{2}+y_{3}}{3}) \end{array}$$

For further analysis, facial data from the last 30 s were extracted and analyzed. That is, we defined the first 180 s as time for the visual content to sufficiently “sink in” for the participants.

### 3.2. Feature Variable Extraction

To extract feature variables involving facial micromovement, we developed a micromovement extraction program built by LabVIEW 2016 for massive data processing. From the last 30 s of the participant’s dataset, 11 AUs (Table 1) were calculated. A threshold was used, the average movement of an AU, using the following min-max algorithm to determine the onset of facial expressions. The micromovement section before the onset was extracted.

$$Threshold=\frac{\left(Max+Min\right)}{2}$$

The expression section after the onset consisted of one macromovement section (4 s) and two micromovement sections (1 s, 0.5 s). These three sections may overlap. The movement data from the three sections were extracted. That is, the degree of change (delta) in the coordination of an AU between the current and previous frames was computed as follows, which was performed to analyze the degree of facial vibration.

$$\begin{array}{l} x_{n}=prevAU[n]\cdot x-currAU[n]\cdot x\\ y_{n}=prevAU[n]\cdot y-currAU[n]\cdot y \end{array}$$

Finally, we extracted feature values by analyzing the delta value. That is, the average and standard deviation of the delta and FFT values were extracted. The former two were used to analyze the degree and variance of the change. The latter was used to analyze the degree of facial vibration through the dominant frequency obtained by the FFT.

### 3.3. Heart Rate Variability Analysis

In addition to the facial data, ECG data were measured while the visual stimuli were shown for 210 s. The participants’ time-series data were transformed into a frequency band using FFT. This enabled measurement of the ANS responses of participants exposed to emotion-inducing stimuli [30,31]. Table 2 outlines the HRV variables used in this study. To measure the change in the serial heart rate data, a 180-s sliding window was used.

## 4. Results

The current study analyzed changes in facial micromovements between real and fake expressions of representative emotions. A *t*-test was used to compare the differences between the participants’ facial expressions in the two conditions. The feature variables obtained at 4 s (macromovement), 1 s, and 0.5 s (micromovements) after the onset (*t*) of facial expression were analyzed.

**Figure 6:**
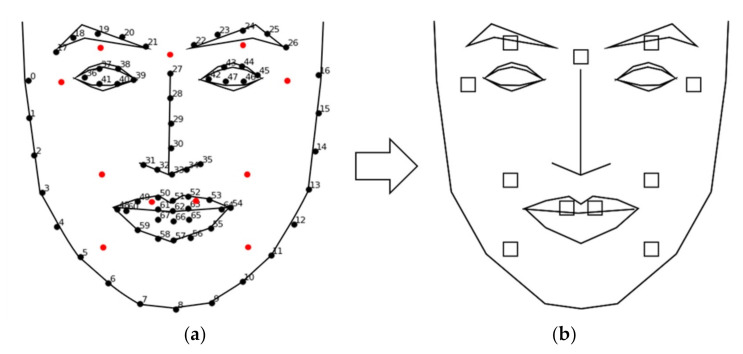
(**a**) Eleven AU regions (red dots) for feature variable extraction; (**b**) visualization framework for reporting the results.

**Figure 7:**
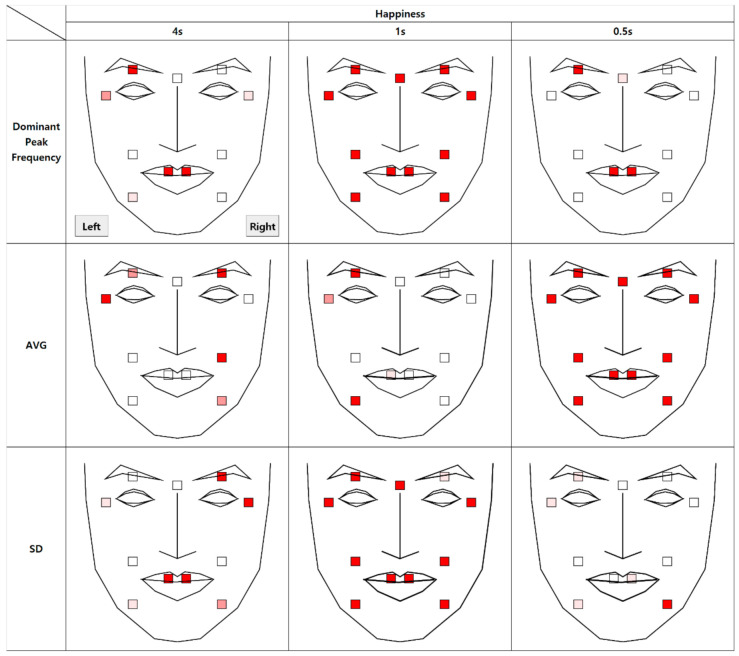
Statistical differences between real and fake happiness expressions (AVG = Average, SD = Standard Deviation).

Figure 6 shows the template used to visualize the results. The blank squares on the right indicate the 11 AUs (Table 1) representing the facial muscles responsible for facial expressions. The statistical difference between the real and fake conditions are color coded in Figures 7, 9, 11 and 13 in three levels: *p* < 0.001: *** 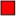; *p* < 0.01: ** 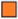; *p* < 0.05: * 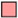.

In the HRV analysis, we compared the difference in ANS activation between the real and fake conditions.

### 4.1. Authenticity of Happiness

The results of the analysis of micromovement involving expressions of happiness are as follows. Figure 7 depicts the differential movement of the facial regions between the two conditions through the visualization of a face. All 11 AUs had at least one significant difference in the dependent variables (dominant peak frequency, average, and standard deviation of movement).

The average at t + 0.5 (0.5 s after the onset) showed a significant difference in all AUs, whereas only partially significant differences appeared at t + 1, mostly in the left face. This implies that expressions of happiness may be most prominent in the early stage (0.5 s) of a microexpression but persist until t + 1 in the left face. Further regression analysis on average movement showed that the time segment factor enters the regression equation (R^2^ = 0.97), *p* < 0.001, along with the authenticity factor, *p* < 0.05.

However, for the standard deviation, the values at t + 1 significantly differed in all 11 AUs. The domain peak frequency also showed a significant difference at t + 1 in all AUs. The domain peak frequency at t + 0.5 showed a significant difference in the lips, left eyebrows, and brow.

**Figure 8:**
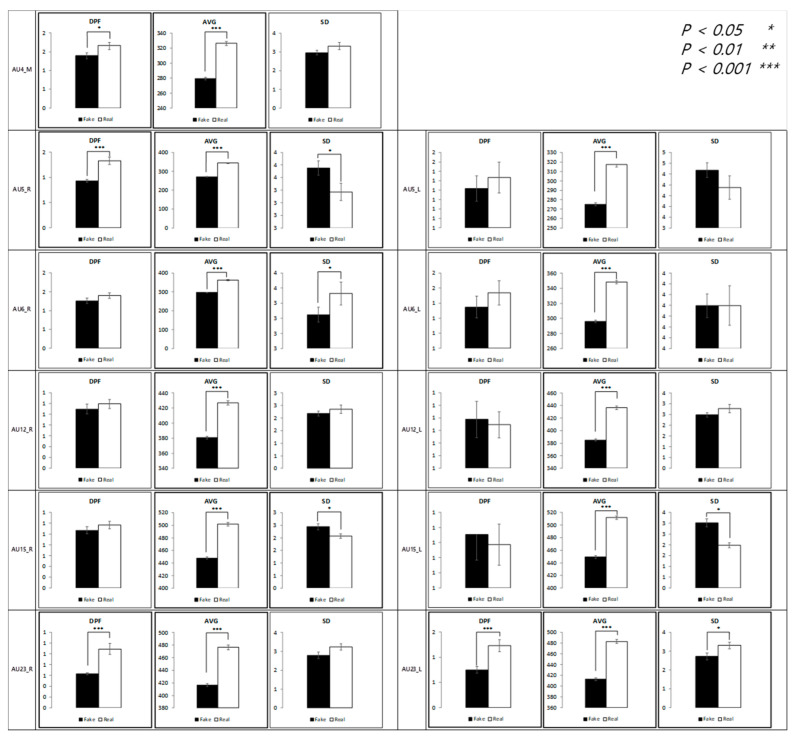
Comparison between feature variables of happiness expressions.

Figure 8 presents a statistical comparison between dependent variables for each AU, collapsing data from the three sections (t + 0.5, t + 1, and t + 4). The measured values were higher in real expressions in almost all regions.

### 4.2. Authenticity of Contentment

**Figure 9:**
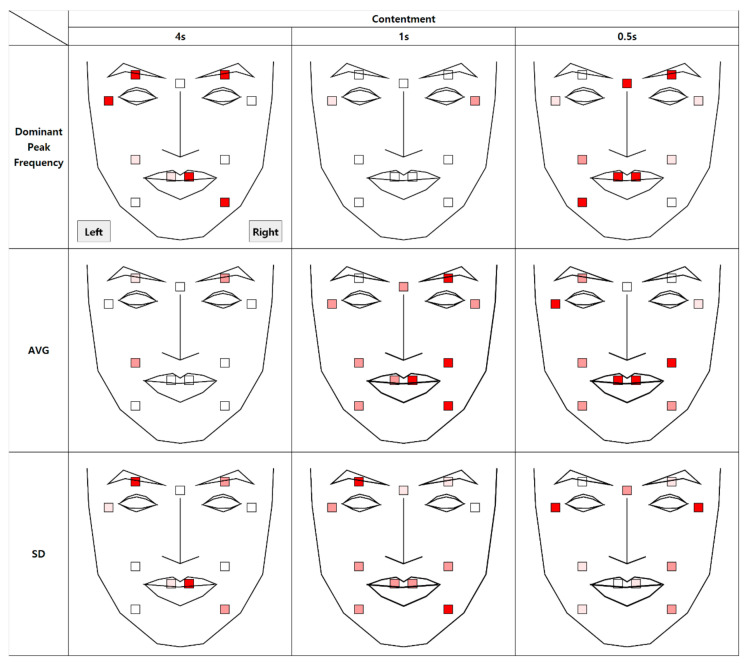
Statistical differences between real and fake contentment expressions (AVG = Average, SD = Standard Deviation).

The results of the analysis of micromovements involving expressions of contentment are as follows. Figure 9 depicts the differences in the movement of facial regions between the two conditions.

At t + 1, except for the left eyelid, all 10 AUs were found to have a significant average difference. Similar results were observed for the standard deviation. At t + 0.5, nine AUs were reported to have a significant average difference. This indicates that the microexpression of contentment, compared to happiness, may persist longer. Further regression analysis on average movement showed that the time segment factor enters the regression equation (R^2^ = 0.97), *p* < 0.001, along with the authenticity factor, *p* < 0.001 and the face side factor, *p* < 0.001.

The vibration of the macromovement (dominant peak frequency at t + 4) was significantly different in many facial regions, including the mouth tail and eyelid of the right side and the eye tail, eyelid, and mouth tail of the left side. Similar results were observed for the standard deviation in the same regions.

**Figure 10:**
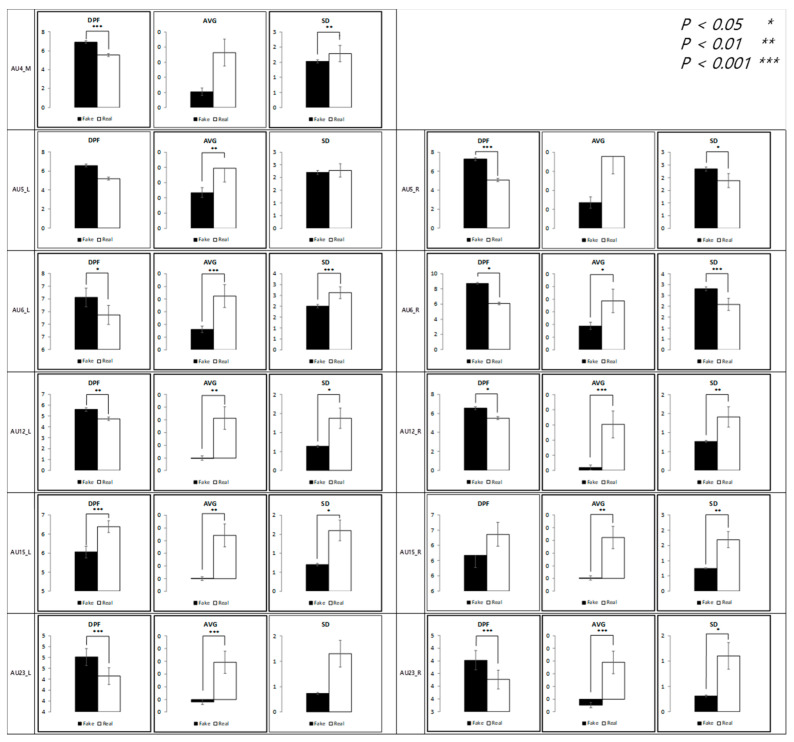
Comparison between feature variables of contentment expressions.

As shown in Figure 10, similar to the happiness condition, the average was significantly higher in the real condition, but the dominant peak frequency was significantly higher in the fake condition. That is, there was more facial movement in the real condition but more facial vibration in the fake condition.

### 4.3. Authenticity of Anger

**Figure 11:**
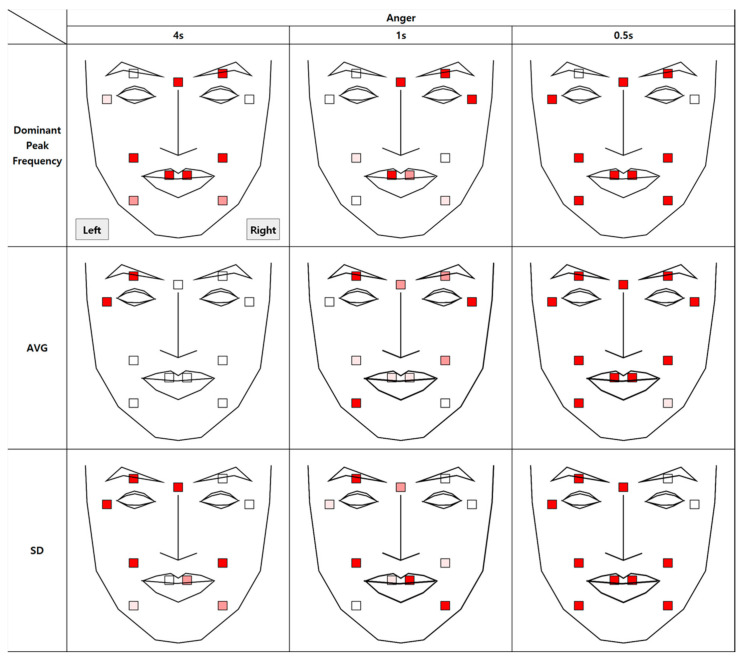
Statistical differences between real and fake anger expressions (AVG = Average, SD = Standard Deviation).

The results of the analysis of micromovement involving expressions of anger are as follows. Figure 11 depicts the differences in the movement of facial regions between the two conditions.

Similar to the results in the happiness condition, micromovements at t + 0.5 had a statistical difference in all regions, 11 of them at *p* < 0.001. Unlike with happiness, however, the differential micromovements of anger persisted through t + 1, except for in two of the facial regions. Further regression analysis on average movement showed that the time segment factor entered the regression equation (R^2^ = 0.96), *p* < 0.001, along with the authenticity factor, *p* < 0.001 and the face side factor, *p* < 0.05.

A significant difference in dominant peak frequency was found in all regions except for the right eye tail and the left eyelid in all time segments.

**Figure 12:**
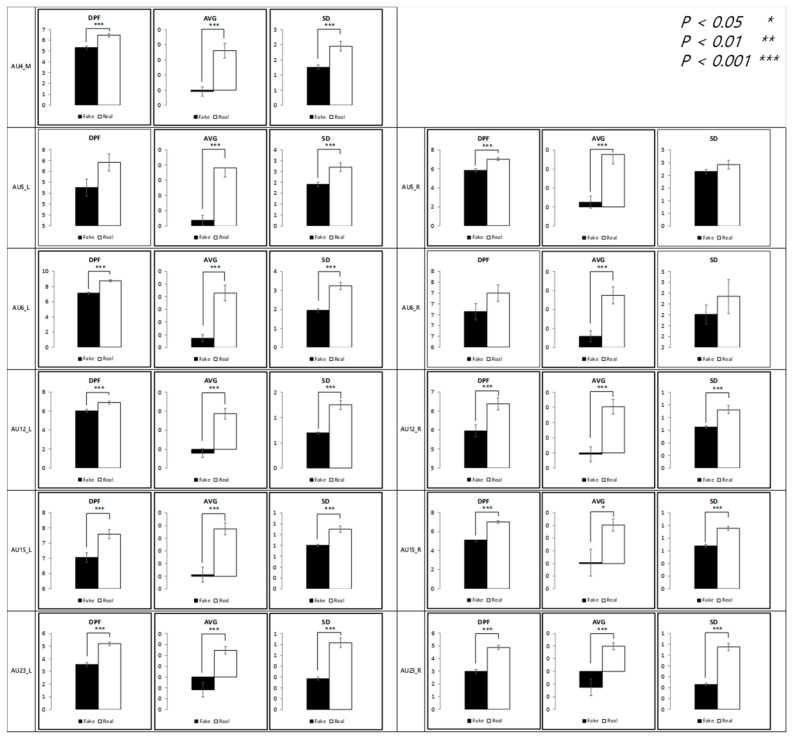
Comparison between feature variables of anger expression.

As shown in Figure 12, similar to the happiness condition but unlike the contentment condition, all three measurements were higher in the real condition than in the fake condition.

### 4.4. Authenticity of Sadness

**Figure 13:**
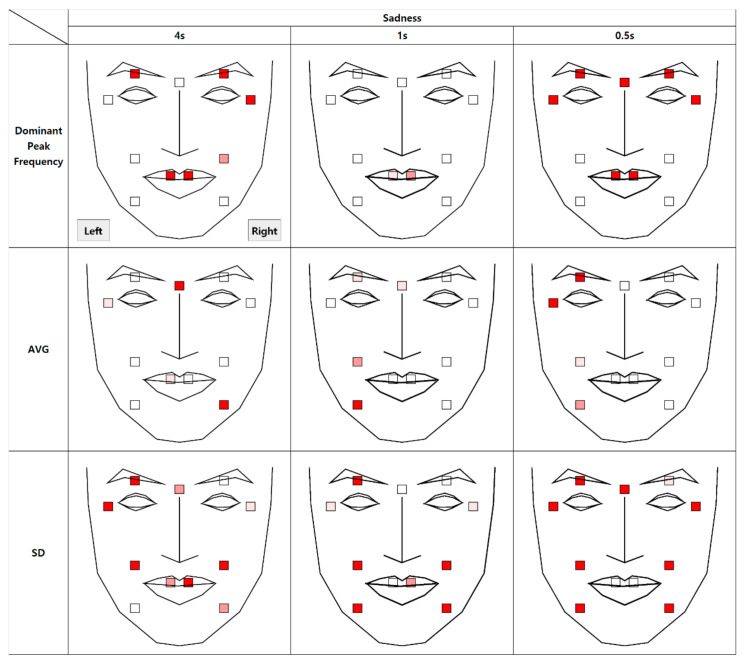
Statistical differences between real and fake sadness expression (AVG = Average, SD = Standard Deviation).

The results of the analysis of micromovement involving the expression of sadness are as follows. Figure 13 depicts the differential movement of the facial regions between the two conditions.

The significant differences were not dominant in all facial regions compared to other emotion conditions, but instead concentrated on the left side of the face. Specifically, similar results were found in the micromovements (t + 1 and t + 0.5) in the left eyelid and mouth tail. Further regression analysis on average movement showed that the face side (left or right) factor enters the regression equation (R^2^ = 0.96), *p* < 0.001, along with the authenticity factor, *p* < 0.001 and the time segment factor, *p* < 0.001.

A significant difference was found in the mouth region in all segments with respect to the dominant peak frequency. However, the difference in vibration was prominent and salient at t + 4 and t + 0.5.

**Figure 14:**
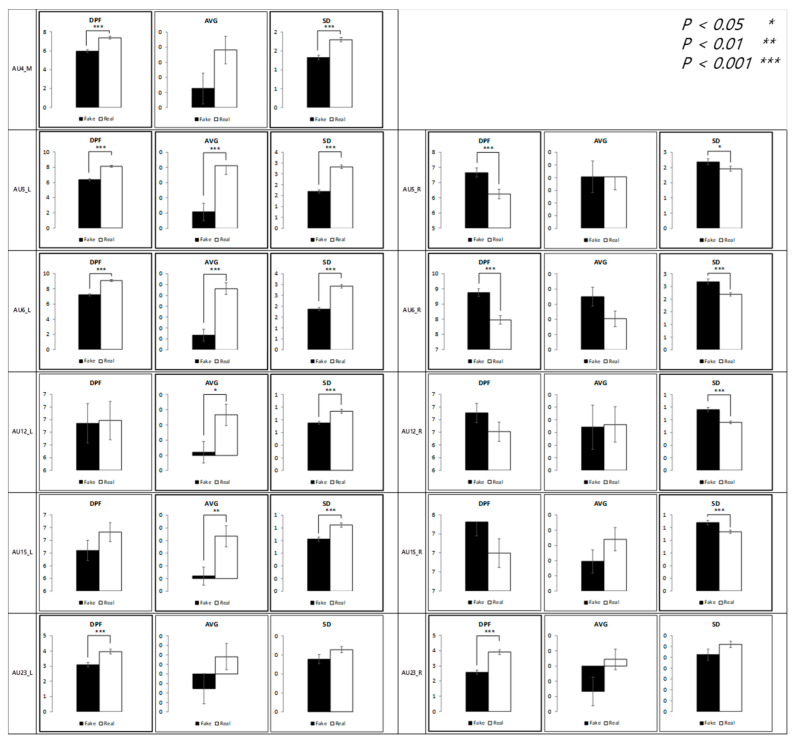
Comparison between feature variables of sadness expressions.

As shown in Figure 14, when the data are collapsed, similar to the contentment condition, the standard deviation and the dominant peak frequency were higher in the fake condition than in the real condition.

### 4.5. Analysis of Heart Rate Variability

**Figure 15:**
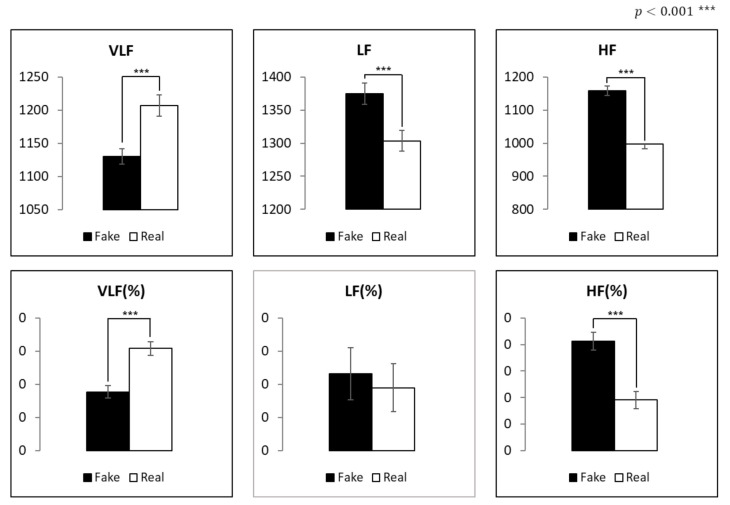
Comparison of frequency domain.

The HRV data of the fake condition were compared to those of the real condition of the three frequency bands (very low, low, and high) (see Figure 15). This was performed to compare the ANS response, independent of emotions. Except for the LF (%) variable, a significant difference was found in all variables (*p* < 0.001). Specifically, VLF and VLF (%) were higher in the real condition than in the fake condition. Conversely, HF and HF (%) were higher in the fake condition than in the real condition. LF was significantly higher in the fake condition.

## 5. Conclusions and Discussion

The present study compared the differences in the micromovement of facial expressions between real and fake emotions. The study utilized 11 AUs based on anatomical muscle location responsible for emotional expression. That is, we identified the difference in the feature variables (average and standard deviation of movement, as well as dominant peak frequency) between the real and fake conditions by facial regions for each representative emotion (happiness, contentment, anger, and sadness). In conclusion, the study showed that the degree of activation is higher if the expression is authentic, implying more micromovement.

The study analyzed the feature variables in three time segments (0.5, 1, and 4 s) after the onset (*t*) of facial expression for each representative emotion. Results indicated that micromovements are more informative at an early stage (less than a second) of expression. In the case of t + 1 and t + 0.5, a significant difference between the real and fake conditions was observed in the left face than the right in the happiness condition. The asymmetric difference in the activation of the face can be explained by activation of the right brain region [32]. Campbell found that the left face expresses more than the right in voluntary expressions. Conversely, the left face expresses less than the right in involuntary expressions [33]. In the anger condition, compared with other emotions, the brow had the highest number of feature variables that were significantly different between the real and fake conditions. This was a result of muscle movement from the participant’s frowning.

At t + 4, compared to the time segments in which less than a second had elapsed, less statistical differences were observed between the two conditions for all four emotions. This confirms that measurements at t + 4 cannot reliably capture the differential micromovements between real and fake expressions. The data at t + 4 also include the macromovements of facial muscles and hence may not be sensitive enough to identify abrupt changes in facial movements (i.e., micromovements).

Collapsing the data across time segments, all three feature variables (average, standard deviation, and dominant peak frequency) of the real condition were significantly higher than those of the fake condition in the happiness and anger conditions. Conversely, in the contentment and sadness conditions, the standard deviation and dominant peak frequency of the fake condition were significantly higher. That is, emotions that are primarily expressed with the relaxation of facial muscles, such as contentment and sadness, were observed with weaker intensity in the real condition. The results support the hypothesis that the degree of expression differs between the real and fake conditions as a function of emotions.

Our findings were cross-validated with neurological measurements involving the PSNS and ANS. In the HRV analysis, both HF and HF (%) indicators for the parasympathetic nervous system (PSNS) were higher in the fake condition than in the real condition. Conversely, both VLF and VLF (%) indicators for the ANS were higher in the real condition than in the fake condition. LF (%), an indicator that involves both the PSNS and ANS, did not show a significant difference. In conclusion, the stimuli in the real condition led to the activation of the ANS, which implies an increase in the participant’s arousal. In addition, the stimuli in the fake condition led to the activation of the PSNS, which implies the participant’s relaxation.

The study acknowledges the individual variance in participants’ emotions when they were exposed to visual stimuli. To minimize this difference, a target facial expression was provided. In the fake condition, to ensure that other emotions did not interfere, visual content inducing a neutral emotion was used. That is, participants had to pretend an expression while the stimuli conveyed neutrality. We acknowledge the limitations of this experimental design, which may lower the ecological validity. However, future studies may investigate when a real emotion is replaced by another emotion and study the change in microexpressions.

Follow-up studies may introduce experimental treatments that are congruent with real-world settings. Specifically, micromovements of expressions in complex emotions merit further analysis. In addition, the study was limited to four representative emotions. Although not related to emotion authenticity, Adegun and Vadapalli analyzed microexpressions to recognize seven universal emotions with machine learning [34].

Another limitation of the study involves facial landmark detection. Proper landmark detection is necessary to secure recognition accuracy [20]. We have identified 68 facial landmarks by tracking the eye, eyebrows, nose, lips, and chin line using the Dlib library [29]. However, recent state-of-the-art methods, including tweaked convolutional neural networks (TCNN), may improve the robustness of facial landmark detection [35].

The breakdown of feature variables may be used as an appraisal criterion to authenticate facial data with emotional expressions. This study identified that data at less than one second is critical for analysis of the authenticity of an expression, which may not be reportable by the participants.

Systems capable of recognizing human emotions (e.g., social robots, recognition systems for the blind, monitoring systems for drivers) may use the authenticity of the user’s facial expression to provide a useful and practical response. Recognizing fake expressions is imperative in security interfaces and systems that counter crime. For a social robot to provide effective services, identifying the user’s intent is paramount. A recent human-robot interaction study applied deep neural networks to recognize a user’s facial expressions in real time [36]. Further recognition of the user’s false (e.g., deception) or pretended (e.g., to be polite) expressions might introduce more social, rich, and effective interactions.

## Figures and Tables

**Table 1:** Action Unit Definitions.

Action Units	Description	Muscular Basis	Landmark
AU4_M	Brow depressor	Depressor glabella	21, 22, 27
		Depressor supercilii	
		Corrugator supercilii	
AU5_L	Upper Lip raiser	Lavator palpebrae superioris	23, 25, 44
AU5_R			18, 20, 37
AU6_L	Cheek raiser	Orbicularis oculi	15, 26, 45
AU6_R	Lip tightener		1, 17, 36
AU12_L	Lip corner puller	Zygomaticus major	14, 35, 54
AU12_R			2, 31, 48
AU15_L	Lip corner depressor	Depressor anguli oris	10, 11, 54
AU15_R			5, 6, 48
AU23_L	Lip tightener	Orbicularis oris	52, 53, 63
AU23_R			49, 50, 61

**Table 2:** Heart Rate Variability Variables.

Variable	Unit	Definition	Frequency Range
VLF	ms^2^	The power value in the VLF frequency	0.003~0.04 Hz
LF	ms^2^	The power value in the LF frequency	0.04~0.15 Hz
HF	ms^2^	The power value in the HF frequency	0.15~0.4 Hz
VLF (%)	%	VLF divided by the overall power value	
LF (%)	%	LF divided by the overall power value	
HF (%)	%	HLF divided by the overall power value	

## Supplementary Materials

The following are available online at https://www.mdpi.com/article/10.3390/s21134616/s1, Python Code_AU Extraction.
