# Supplementary material for: The Analysis of Emotion Authenticity Based on Facial Micromovements
Source: Sensors (Basel). 2021 Jul 5;21(13):4616. doi: 10.3390/s21134616 (PMC8271774; doi:10.3390/s21134616)
Supplement: Supplementary file 1 [file sensors-21-04616-s001.zip › sensors-1203997-supplementary.pdf]

```python
import cv2

import dlib

import numpy as np

import os


# Detect face

def detect_roi_bound(frame, face_cascade, minSize=(150, 150)):

    # Detect face

    faces = face_cascade.detectMultiScale(

        frame,

        scaleFactor=1.1,

        minNeighbors=1,

        # minSize=(100, 100),

        minSize=minSize,

    )

    # Detect roi which have max area/ We are only interested in the 'largest' face

    # So, we determine this based on the largest area of the found rectangle.

    # First initialize the required variables to 0

    max_area = 0

    roi = (0, 0, 0, 0)

    is_detect = False

    # Loop over all faces and check if the area for this face is the largest so far

    for i in range(len(faces)):

        area = faces[i][0] * faces[i][1]

        if area > max_area:

            max_area = area

            roi = faces[i]

        is_detect = True

    #return (630, 320, 300, 300), True

    #print("Optimal", roi)

    return roi, is_detect


# Detect face

def detect_roi_bounds(frame, face_cascade):
```

```
    # Detect face
    faces = face_cascade.detectMultiScale(
        frame,
        scaleFactor=1.1,
        minNeighbors=1,
        minSize=(150, 150),
    )
    # Detect roi from first face
    rois = []
    is_detect = False
    for (x, y, w, h) in faces:
        roi = (x, y, w, h)
        rois.append(roi)
        is_detect = True

    #return [(630, 320, 300, 300)], True
    #print("First ", rois, is_detect)
    return rois, is_detect

def define_facial_landmark_points(predictor, frame, roi):        # predictor: dlib model
    shape = predictor(frame, roi)
    return shape

def shape_to_np(shape, dtype="int"):
    # initialize the list of (x, y)-coordinates to zero
    coords = np.zeros((68, 2), dtype=dtype)

    # loop over the 68 facial landmarks and convert them
    # to a 2-tuple of (x, y)-coordinates
    for i in range(0, 68):
        coords[i] = (shape.part(i).x, shape.part(i).y)
    # return the list of (x, y)-coordinates
    return coords
```

```
# au centroid calculation
def calculate_au_centroid(coords, dtype="int"):
    # define au points
    # x = round((x1 + x2 + x3) / 3, 2)
    # y = round((y1 + y2 + y3) / 3, 2)
    au_list = np.zeros((13, 2), dtype=dtype)

    M_au4_x = round((coords[21][0] + coords[22][0] + coords[27][0]) / 3, 2)
    M_au4_y = round((coords[21][1] + coords[22][1] + coords[27][1]) / 3, 2)

    R_au5_x = round((coords[18][0] + coords[20][0] + coords[37][0]) / 3, 2)
    R_au5_y = round((coords[18][1] + coords[20][1] + coords[37][1]) / 3, 2)
    R_au6_x = round((coords[1][0] + coords[17][0] + coords[36][0]) / 3, 2)
    R_au6_y = round((coords[1][1] + coords[17][1] + coords[36][1]) / 3, 2)
    R_au12_x = round((coords[2][0] + coords[31][0] + coords[48][0]) / 3, 2)
    R_au12_y = round((coords[2][1] + coords[31][1] + coords[48][1]) / 3, 2)
    R_au15_x = round((coords[5][0] + coords[6][0] + coords[48][0]) / 3, 2)
    R_au15_y = round((coords[5][1] + coords[6][1] + coords[48][1]) / 3, 2)
    R_au23_x = round((coords[49][0] + coords[50][0] + coords[61][0]) / 3, 2)
    R_au23_y = round((coords[49][1] + coords[50][1] + coords[61][1]) / 3, 2)

    L_au5_x = round((coords[23][0] + coords[25][0] + coords[44][0]) / 3, 2)
    L_au5_y = round((coords[23][1] + coords[25][1] + coords[44][1]) / 3, 2)
    L_au6_x = round((coords[15][0] + coords[26][0] + coords[45][0]) / 3, 2)
    L_au6_y = round((coords[15][1] + coords[26][1] + coords[45][1]) / 3, 2)
    L_au12_x = round((coords[14][0] + coords[35][0] + coords[54][0]) / 3, 2)
    L_au12_y = round((coords[14][1] + coords[35][1] + coords[54][1]) / 3, 2)
    L_au15_x = round((coords[10][0] + coords[11][0] + coords[54][0]) / 3, 2)
    L_au15_y = round((coords[10][1] + coords[11][1] + coords[54][1]) / 3, 2)
    L_au23_x = round((coords[52][0] + coords[53][0] + coords[63][0]) / 3, 2)
    L_au23_y = round((coords[52][1] + coords[53][1] + coords[63][1]) / 3, 2)

    au_list[0] = (M_au4_x, M_au4_y)
    au_list[1] = (L_au5_x, L_au5_y)
```

```
    au_list[2] = (L_au6_x, L_au6_y)
    au_list[3] = (L_au12_x, L_au12_y)
    au_list[4] = (L_au15_x, L_au15_y)
    au_list[5] = (L_au23_x, L_au23_y)
    au_list[6] = (R_au5_x, R_au5_y)
    au_list[7] = (R_au6_x, R_au6_y)
    au_list[8] = (R_au12_x, R_au12_y)
    au_list[9] = (R_au15_x, R_au15_y)
    au_list[10] = (R_au23_x, R_au23_y)

    return au_list


def track_roi_straight_forward(roi, prev_roi):
    x, y, w, h = roi
    prev_x, prev_y, prev_w, prev_h = prev_roi
    if (x < prev_x + prev_w) & \
        (x + w > prev_x) & \
        (y < prev_y + prev_h) & \
        (y + h > prev_y):
        return True
    return False


def extract_raw_signal_from_facial_landmark_points(prev_shape, curr_shape):
    raw_signal_x = []
    raw_signal_y = []

    for prev_s, curr_s in zip(prev_shape, curr_shape):
        x = prev_s[0] - curr_s[0]
        y = prev_s[1] - curr_s[1]
        raw_signal_x.append(x)
        raw_signal_y.append(y)
    #print("landmark", raw_signal_x)
    return raw_signal_x, raw_signal_y
```

```
def extract_raw_signal_from_facial_au_points(prev_au_shape, curr_au_shape):
    au_signal_x = []
    au_signal_y = []

    for prev_au, curr_au in zip(prev_au_shape, curr_au_shape):
        au_x = prev_au[0] - curr_au[0]
        au_y = prev_au[1] - curr_au[1]
        au_signal_x.append(au_x)
        au_signal_y.append(au_y)
    #print("au", au_signal_x)
    return au_signal_x, au_signal_y


def calculate_size_diff(roi, prev_roi):
    _, _, w, h = roi
    _, _, prev_w, prev_h = prev_roi
    roi_size = w * h
    prev_roi_size = prev_w * prev_h
    size_diff = abs(roi_size - prev_roi_size)
    return size_diff


def extract_signal_straight_forward(frames, haar_path='./haarcascade_frontalface_alt2.xml', dlib_path='./shape_predictor_68_face_landmarks.dat', is_display=False):
    # Load face cascade
    face_cascade = cv2.CascadeClassifier()
    is_load = face_cascade.load(haar_path)
    print('Load cascade ', is_load)

    # Load shape predictor
    predictor = dlib.shape_predictor(dlib_path)

    # Initialize variables
    is_init = False
    is_au_init = False
    is_detect = False
```

```
roi = None
prev_roi = None
prev_shape = None
prev_au_shape = None
signals_x = [[] for _ in range(68)]
signals_y = [[] for _ in range(68)]
au_signals_x = [[] for _ in range(13)]
au_signals_y = [[] for _ in range(13)]


# Loop
for frame in frames:
    try:
        # Convert to gray scale
        gray_frame = cv2.cvtColor(frame, cv2.COLOR_BGR2GRAY)
        # 1. ROI detection - Viola-Jones
        if is_detect is False:
            roi, is_detect = detect_roi_bound(gray_frame, face_cascade=face_cascade, minSize=(150, 150))
            if is_detect is False:
                print('Detection is failed')
                for i in range(68):
                    signals_x[i].append(0)
                    signals_y[i].append(0)
                continue
        # 3. ROI tracking - Straight forward
        else:
            is_track = False
            rois, _ = detect_roi_bounds(gray_frame, face_cascade=face_cascade)
            min_size_diff = None
            for i in range(len(rois)):
                is_track = track_roi_straight_forward(rois[i], prev_roi)
                if is_track:
                    size_diff = calculate_size_diff(rois[i], prev_roi)
                    if min_size_diff is None:
```

```
                roi = rois[i]
                min_size_diff = size_diff
            elif min_size_diff > size_diff:
                roi = rois[i]
                min_size_diff = size_diff
    if is_track is False:
        roi = prev_roi

# 2. ROI definition - Facial landmark point
roi_dlib = dlib.rectangle(int(roi[0]), int(roi[1]), int(roi[0] + roi[2]), int(roi[1] + roi[3]))
shape = define_facial_landmark_points(predictor, gray_frame, roi_dlib)
shape = shape_to_np(shape)
shape_au = calculate_au_centroid(shape)

#print(shape)
#print(shape_au)

# 4. Raw signal extraction - Head motion based method
if is_init:
    signal_x, signal_y = extract_raw_signal_from_facial_landmark_points(prev_shape, shape)
    for i in range(68):
        signals_x[i].append(signal_x[i])
        signals_y[i].append(signal_y[i])
else:
    is_init = True


#5. Raw signal extraction - au piont - Head motion based method
if is_au_init:
    au_signal_x, au_signal_y = extract_raw_signal_from_facial_au_points(prev_au_shape, shape_au)
    for i in range(13):
        au_signals_x[i].append(au_signal_x[i])
        au_signals_y[i].append(au_signal_y[i])
```

```
            else:
                is_au_init = True



            # Display
            if is_display is True:
                cv2.imshow('raw_frame', frame)
                cv2.rectangle(frame, (roi[0], roi[1]), (roi[0] + roi[2], roi[1] + roi[3]), (0, 255, 0), 3, 4, 0)
                for (x, y) in shape:
                    cv2.circle(frame, (x, y), 1, (0, 0, 255), -1)
                for (au_x, au_y) in shape_au:
                    cv2.circle(frame, (au_x, au_y), 1, (255, 0, 0), -1)
                cv2.imshow('frame', frame)

                k = cv2.waitKey(30) & 0xff
                if k == 30:
                    break

            # Save previous roi
            prev_roi = roi
            prev_shape = shape
            prev_au_shape = shape_au

        except:
            break

    return signals_x, signals_y, au_signals_x, au_signals_y

def test_cam_straight_forward(result_filename, haar_path='./haarcascade_frontalface_alt2.xml', dlib_path='./shape_predictor_68_face_landmarks.dat'):
    import cv2

    # Open file
    f = open('./' + result_filename + '.txt', 'w')
```

```
# Open video
cap = cv2.VideoCapture(0)

# Load face cascade
face_cascade = cv2.CascadeClassifier()
is_load = face_cascade.load(haar_path)
print('Load cascade ', is_load)

# Load shape predictor
predictor = dlib.shape_predictor(dlib_path)

# Initialize variables
is_init = False
is_detect = False
roi = None
prev_roi = None
prev_shape = None
signals_x = [[] for _ in range(68)]
signals_y = [[] for _ in range(68)]

# Read frames
while True:
    try:
        ret, frame = cap.read()    # Capture frame
        if ret:
            try:
                # Convert to gray scale
                gray_frame = cv2.cvtColor(frame, cv2.COLOR_BGR2GRAY)
                # 1. ROI detection - Viola-Jones
                if is_detect is False:
                    roi, is_detect = detect_roi_bound(gray_frame, face_cascade=face_cascade)
                    if is_detect is False:
                        print('Detection is failed')
```

```
                f.write(str(0) + '\n')
                continue
        # 3. ROI tracking - Straight forward
        else:
            is_track = False
            rois, _ = detect_roi_bounds(gray_frame, face_cascade=face_cascade)
            for i in range(len(rois)):
                is_track = track_roi_straight_forward(rois[i], prev_roi)
                if is_track:
                    roi = rois[i]
                    break
            if is_track is False:
                roi = prev_roi

        # 2. ROI definition - Facial landmark point
        roi_dlib = dlib.rectangle(int(roi[0]), int(roi[1]), int(roi[0] + roi[2]), int(roi[1] + roi[3]))
        shape = define_facial_landmark_points(predictor, gray_frame, roi_dlib)
        shape = shape_to_np(shape)
        print('shape=', shape)

        # 4. Raw signal extraction - Head motion based method
        if is_init:
            signal_x, signal_y = extract_raw_signal_from_facial_landmark_points(prev_shape, shape)
            for i in range(68):
                signals_x[i].append(signal_x[i])
                signals_y[i].append(signal_y[i])
            print('signal=', signal_x, signal_y)
            # Write signal to file
            for i in range(68):
                f.write(str(signal_x[i]) + '\t')
            for i in range(68):
                f.write(str(signal_y[i]) + '\t')
            f.write('\n')
        else:
```

```
                    is_init = True

                # Display
                cv2.rectangle(frame, (roi[0], roi[1]), (roi[0] + roi[2], roi[1] + roi[3]), (0, 255, 0), 3, 4, 0)
                for (x, y) in shape:
                    cv2.circle(frame, (x, y), 1, (0, 0, 255), -1)
                cv2.imshow('frame', frame)

                k = cv2.waitKey(30) & 0xff
                if k == 30:
                    break

                # Save previous roi
                prev_roi = roi
                prev_shape = shape

            except:
                break
        else:
            break
    except Exception as e:
        print(e)
        break

# Close file
cap.release()
f.close()
```

```
def test_video_straight_forward(filename, landmark_filename, au_filename, haar_path='./haarcascade_frontalface_alt2.xml', dlib_path='./shape_predictor_68_face_landmarks.dat'):
    import cv2

    # Open file
```

```
    f1 = open('./' + landmark_filename + '.txt', 'w')
    f2 = open('./' + au_filename + '.txt', 'w')

    # Open video
    cap = cv2.VideoCapture(filename)

    # Read frames
    frames = []
    while True:
        try:
            ret, frame = cap.read()    # Capture frame
            if ret:
                frames.append(frame)
            else:
                break
        except:
            break

    # Close video
    cap.release()

    # Extract signal
    signal_x, signal_y, au_signal_x, au_signal_y = extract_signal_straight_forward(frames, haar_path=haar_path, dlib_path=dlib_path, is_display=True)



    # Write signal to file_landmark
    for i in range(len(signal_x[0])):
        for j in range(len(signal_x)):
            f1.write(str(signal_x[j][i]) + "\t")
        for j in range(len(signal_x)):
            f1.write(str(signal_y[j][i]) + "\t")
        f1.write('\n')
```

```
    # Write signal to file_au
    for i in range(len(au_signal_x[0])):
        for j in range(len(au_signal_x)):
            f2.write(str(au_signal_x[j][i]) + "\t")
        for j in range(len(au_signal_x)):
            f2.write(str(au_signal_y[j][i]) + "\t")
        f2.write('\n')
    # for s in signal:
    #     f.write(str(s) + '\n')

    # Close file
    f1.close()
    f2.close()

def load_data():
    import os
    dir = 'D:/1. Project/RFSmile/Real/Real_2'
    #dir = 'D:/1. Project/RFSmile/Fake/Fake_2'

    for root, dirs, files in os.walk(dir):
        for file in files:
            user_name = file[:-8]
            filename = dir + '/' + file
            # analysis(filename)
            test_video_straight_forward(filename,
                                        landmark_filename= user_name +'_landmark',
                                        au_filename= user_name + '_au')

if __name__ == '__main__':
    #test_cam_straight_forward(result_filename='v_sw')
    path_dir = 'D:/1. Project/RFSmile/Real/Real_2'
    user_name = '/Ohsojung_Real_1_cut'

    test_video_straight_forward(filename=path_dir + user_name + '.mp4',
```

```
landmark_filename= user_name +'_landmark',
au_filename= user_name + '_au')

#load_data()
```
